# Towards Precision Therapies for Inherited Disorders of Neurodegeneration with Brain Iron Accumulation

**DOI:** 10.5334/tohm.661

**Published:** 2021-11-24

**Authors:** Robert V.V. Spaull, Audrey K.S. Soo, Penelope Hogarth, Susan J. Hayflick, Manju A. Kurian

**Affiliations:** 1 Developmental Neurosciences, Zayed Centre for Research into Rare Disease in Children, UCL Great Ormond Street Institute of Child Health, UCL, London, UK; 2 Department of Paediatric Neurology, Great Ormond Street Hospital for Children, London, UK; 3 NIHR Great Ormond Street Biomedical Research Centre, London, UK; 4 Departments of Molecular & Medical Genetics and Neurology, Oregon Health & Science University, Portland, Oregon, US

**Keywords:** NBIA, pantothenate kinase-associated neurodegeneration, PKAN, PLAN, MPAN, gene therapy

## Abstract

**Background:**

Neurodegeneration with brain iron accumulation (NBIA) disorders comprise a group of rare but devastating inherited neurological diseases with unifying features of progressive cognitive and motor decline, and increased iron deposition in the basal ganglia. Although at present there are no proven disease-modifying treatments, the severe nature of these monogenic disorders lends to consideration of personalized medicine strategies, including targeted gene therapy. In this review we summarize the progress and future direction towards precision therapies for NBIA disorders.

**Methods:**

This review considered all relevant publications up to April 2021 using a systematic search strategy of PubMed and clinical trials databases.

**Results:**

We review what is currently known about the underlying pathophysiology of NBIA disorders, common NBIA disease pathways, and how this knowledge has influenced current management strategies and clinical trial design. The safety profile, efficacy and clinical outcome of clinical studies are reviewed. Furthermore, the potential for future therapeutic approaches is also discussed.

**Discussion:**

Therapeutic options in NBIAs remain very limited, with no proven disease-modifying treatments at present. However, a number of different approaches are currently under development with increasing focus on targeted precision therapies. Recent advances in the field give hope that novel strategies, such as gene therapy, gene editing and substrate replacement therapies are both scientifically and financially feasible for these conditions.

**Highlights:**

This article provides an up-to-date review of the current literature about Neurodegeneration with Brain Iron Accumulation (NBIA), with a focus on disease pathophysiology, current and previously trialed therapies, and future treatments in development, including consideration of potential genetic therapy approaches.

## Introduction

Neurodegeneration with brain iron accumulation (NBIA) comprises a group of rare but devastating inherited neurological diseases with unifying features of progressive cognitive and motor decline, and increased iron deposition in the basal ganglia. The most common disorders that present in infancy and childhood are Beta-propeller Protein-Associated Neurodegeneration (BPAN), Pantothenate Kinase-Associated Neurodegeneration (PKAN), PhosphoLipase A_2_-Associated Neurodegeneration (PLAN), and Mitochondrial membrane Protein-Associated Neurodegeneration (MPAN). Several other less common NBIA disorders are also reported, with 15 monogenic causes of neurodegeneration with brain iron accumulation currently proposed. These disorders have the common neuroradiological feature of signal hypointensity of the basal ganglia on specific MR sequences known to demonstrate the magnetic resonance phenomenon of susceptibility which indicates excess mineralization (T2-weighted, T2*-weighted, susceptibility-weighted and echo-planar imaging b0-diffusion imaging data sets) [1] with some disorders also clearly associated with neuropathological findings of iron accumulation on post-mortem analysis. Over time, it is increasingly apparent that such radiological features are associated with a broad spectrum of neurological and neurodegenerative disorders including the mitochondrial cytopathies, genetic dystonias (such as those due to *KMT2B and VPS16* mutations) and lysosomal disorders (GM1 gangliosidosis, alpha fucosidosis) [2,3,4,5], although for many of these disorders, correlation with post-mortem studies is not yet reported. As a result, precise classification of what comprises an NBIA disorder remains uncertain.

Common clinical presentations of NBIA disorders include infantile or childhood onset loss of motor skills and evolution of a movement disorder, often including dystonia and parkinsonism, with cognitive regression and neuropsychiatric involvement. Eye involvement and epilepsy are features of certain disorders, and other organ systems less frequently. MRI findings always include evidence of excess mineralization at least by a certain stage of disease progression, often involving the globus pallidus (GP) and substantia nigra (SN); less frequent MRI findings include white matter changes, and atrophy of the cerebellum or cerebrum.

Diagnosis is often suspected based on typical clinical presentation in conjunction with MRI findings suggestive of iron deposition. Definitive diagnosis requires genetic testing either of single genes if strongly suspected (such as *PANK2* if typical MRI changes are seen), using a panel of genes focused on the clinical or imaging phenotype, or broader next generation whole exome and whole genome sequencing strategies [6]. It is currently estimated that a genetic diagnosis is confirmed in 85% of patients diagnosed clinically and neuroradiologically to have an NBIA disorder [7].

In tandem with the genetic revolution, there have been advances in the development of targeted therapeutic strategies for rare genetic disorders. Indeed, in recent years, previously untreatable childhood-onset neurogenetic conditions have had their first treatments licensed. These include cerliponase alfa enzyme replacement therapy for ceroid lipofuscinosis type 2 (late infantile Batten disease) [8], advances in spinal muscular atrophy treatment with the antisense oligonucleotide treatment nusinersen [9], and targeted gene therapy for aromatic L-amino acid decarboxylase (AADC) deficiency [10,11]. At present there are no proven disease-modifying precision treatments for the NBIA disorders. However, the severity and pharmacoresistant nature of these monogenic conditions render them ideal for similar personalized approaches. In this review we summarize the pathophysiology, current evidence towards disease modifying treatments, and future direction towards precision therapies.

## Methods

In April 2021, the authors used PubMed to perform a literature search of articles from any time using the terms “neurodegeneration with brain iron accumulation” with either “treatment” or “therapy”, then each individual NBIA disorder with the same. This returned 448 results (Supplementary Table 1) and following removal of duplicates and non-English language publications 326 remained. All articles were reviewed for relevance and 88 are referenced in this review. References and co-citations (*CoCites.com*) from these articles were thoroughly searched for additional articles, and a further 36 pertinent articles are included.

In addition, public clinical trials databases (*ClinicalTrials.gov*, EU Clinical Trials Register, and International Clinical Trials Registry Platform) were searched for studies relevant to NBIAs, yielding 21 unique studies – of these, 12 were interventional, 5 completed and 7 reportedly underway or recruiting (***Table 1***).

**Table 1:** **Clinical trial database search results**, June 2021. Results of clinical trials database search for NBIA disorders. All interventional trials, completed and active, found on *ClinicalTrials.gov* or the EU Clinical Trials Register are listed. * Status is as reported on clinical trials database in August 2021 and may not represent actual trial enrolment status.


NBIA	DATABASE REF.	STATUS*	LOCATION

Pilot Study: Iron-chelating treatment in patients with Neurodegeneration with Brain Iron Accumulation (NBIA)	NCT00907283 2008-005206-39	Ongoing	Italy

**PKAN**			

CoA-Z in Pantothenate Kinase-associated Neurodegeneration (PKAN)	NCT04182763	Recruiting	North America

A study for efficacy of pantethine in the treatment of pantothenate kinase-associated neurodegeneration	ChiCTR1900021076	Completed	China

Stimulation of the Globus pallidus internus in patients with NBIA (formerly Hallervorden-Spatz-Syndrome): prospective analysis of international therapeutic outcomes and development of a therapeutic algorithm	DRKS00003106	Recruiting	Worldwide

Compassionate Use of Deferiprone in Patients With PKAN	NCT02635841	Available	–

Efficacy and Safety Study of Fosmetpantotenate (RE-024) in PKAN Participants	NCT03041116 2016-001955-29	Terminated	Europe, USA

Long-term Safety and Efficacy Study of Deferiprone in Patients with Pantothenate Kinase-Associated Neurodegeneration (TIRCON-EXT)	NCT02174848 2014-001427-79	Completed	Germany, UK

A randomized, double-blind, placebo-controlled trial of deferiprone in patients with pantothenate kinase-associated neurodegeneration (TIRCON)	ISRCTN72904618 NCT01741532 2012-000845-11	Completed	Europe, USA

Phase II trial to assess safety and efficacy of Iron chelating agent Deferiprone in patients with Pantothenate Kinase-Associated Neurodegeneration	2008-003059-56	Completed	Italy

**PLAN**			

A Study to Assess Efficacy and Safety of RT001 in Subjects With Infantile Neuroaxonal Dystrophy	NCT03570931	Active, not recruiting	USA

Desipramine in Infantile Neuroaxonal Dystrophy (INAD)	NCT03726996	Terminated	USA

**Aceruloplasminemia**			

Clinical Curative Effect Evaluation Study of Treatment of Oral Deferiprone Tablets in Aceruloplasminaemia Patients	NCT04184453	Recruiting	China


## NBIA Pathophysiology and the role of iron dyshomeostasis

**Figure 1:**
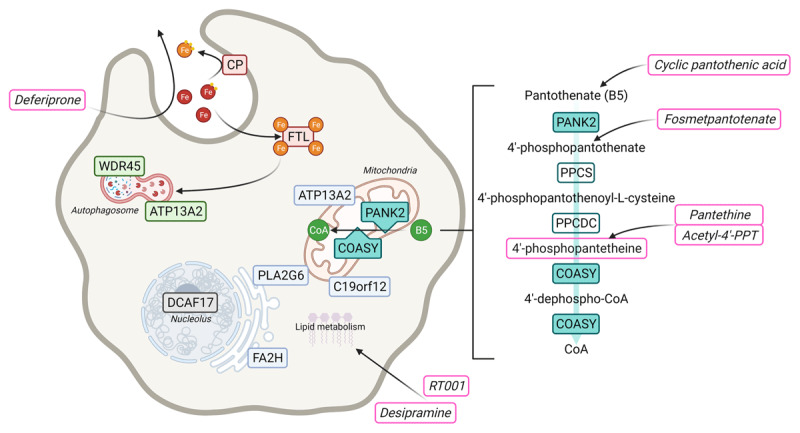
**Overview of the cellular localization of causative genes, implicated pathways, and therapeutic development in monogenic NBIA disorders.** Schematic diagram indicating proposed cellular localization of proteins encoded by NBIA-associated genes and their implicated pathways: CoA biosynthesis (turquoise), iron homeostasis (red), lipid metabolism (blue), autophagy (green), and unknown mechanism (grey). Agents in a pink box indicates treatments trialed or in development. B5, vitamin B5 (pantothenate); CoA, coenzyme A.

The pathophysiology of inherited NBIA disorders is heterogeneous, reflecting that brain iron accumulation may be secondary to a number of different neurodegenerative processes that lead to a common endpoint. There are currently over 10 posited monogenic NBIA disorders (***Table 2***), with causative genes involved in diverse cellular processes, including coenzyme A (CoA) biosynthesis (PKAN, CoPAN), lipid metabolism (PLAN) and autophagy (BPAN) (***Figure 1***). Interestingly, only two NBIA disorders (aceruloplasminemia and neuroferritinopathy) are caused by defects in proteins that are known to be directly associated with iron homeostasis.

**Table 2:** **Summary of reported NBIA genes.** Genes tabulated in order of estimated prevalence, from most commonly identified to least commonly identified NBIA subtypes [124]. Abbreviations: aNAD, atypical neuroaxonal dystrophy; BG, basal ganglia; BPAN, β-propeller protein–associated neurodegeneration; CoA, coenzyme A; CoPAN, CoA synthase protein–associated neurodegeneration; ER, endoplasmic reticulum; FAHN, fatty acid hydroxylase–associated neurodegeneration; GP, globus pallidus; INAD, Infantile Neuroaxonal Dystrophy; MIM, Mendelian Inheritance in Man; MPAN, mitochondrial membrane protein–associated neurodegeneration; MRI, magnetic resonance imaging; NBIA, neurodegeneration with brain iron accumulation; PKAN, pantothenate kinase–associated neurodegeneration; PLAN, phospholipase A2-associated neurodegeneration; 4’-PPT, 4’-phosphopantetheine; RCT, randomized controlled trial; SN, substantia nigra. ‡Mineralization in the brain has specific patterns on brain MRI with iron (Fe3+) accumulation appearing hypointense on T2-weighted images and isointense on T1-weighted images.


NBIA DISORDER MIM NUMBER	GENE INHERITANCE MIM	CELLULAR LOCALIZATION	PROTEIN FUNCTION	MAIN CLINICAL FEATURES OR PHENOTYPES	LOCATION OF MRI MINERALIZATION‡	OTHER MRI FINDINGS	NEUROPATHOLOGY	TRIALED THERAPIES

BPAN #300894	*WDR45* XLD *300526	Autophagosome	Scaffolding protein for assembly of multiprotein complexes	Early childhood onset epilepsy, developmental delay, ataxia, and stereotypies followed by progression with dystonia and parkinsonism in young adulthood	GP, SN	SN has thin, hypointense band surrounded by a ‘halo’ of hyperintensity on T1	GP and SN: iron, gliosis, neuronal loss and spheroids, tau	

PKAN #234200	*PANK2* AR *606157	Mitochondria	Essential regulatory enzyme in CoA biosynthesis	Dystonia, parkinsonism, retinal degeneration Classical: Onset in childhood, non-ambulant by age 10 Atypical: Later onset, more varied progression	GP, can also affect SN and STN	‘Eye of the tiger’ sign in GP: T2 hypointensity with central hyperintensity	GP: iron, gliosis, neuronal loss, and spheroids	Deferiprone (oral iron chelation) – RCT completed Fosmetpantotenate (4’-PPA precursor) – RCT completed Oral pantethine (licensed for hyperlipidemia) single-arm, open-label trial completed 4’-PPT – RCT recruiting

PLAN #610217	*PLA2G6* AR *603604	Mitochondria, cytosol, ER	Enzyme that catalyzes release of fatty acids from phospholipids	INAD: Regression in early infancy, strabismus, nystagmus, axial hypotonia and peripheral spasticity with dystonia and progressive tetraparesis. Possible seizures. aNAD: Onset in childhood, similar phenotype to INAD but slower progressing Dystonia-parkinsonism: Adult-onset	GP, SN (may not be present in early disease)	Cerebellar atrophy and claval hypertrophy in INAD and aNAD	Widespread axonal spheroids, Lewy bodies, neurofibrillary tangles, and tau pathology	Deuterated polyunsaturated fatty acid (RT001) open-label trial completed Oral desipramine (tricyclic antidepressant) open-label trial in terminated

MPAN #614298	*C19orf12* AR/AD *614297	Mitochondria, ER, mitochondria associated membrane	Unknown	Childhood dystonia, pyramidal signs, neuropsychiatric features, cognitive decline	GP, SN	T2-hyperintense streaking between GP interna and externa, cerebellar and cortical atrophy	GP: iron, gliosis, neuronal loss, and spheroids Widespread Lewy bodies SN neuron loss	

Aceruloplasminemia #604290	*CP* AR *117700	Plasma membrane	Peroxidation of ferrous transferrin to ferric transferrin	Adult-onset chorea, tremor, and dystonia. Retinal degeneration, diabetes mellitus, and anemia also occur with systemic iron deposition.	BG, thalamus, dentate, red nucleus	Prominent white matter T2 hyperintensity, cerebellar atrophy	Spheroid bodies in astrocytes and neurons	Deferiprone (oral iron chelation) in open-label trial recruiting

FAHN #612319	*FA2H* AR *611026	ER	Catalyzes the synthesis of 2-hydroxysphingolipids	Childhood dystonia and spasticity	GP, SN	Prominent white matter T2 hyperintensity, pontocerebellar atrophy	Unknown	

Kufor-Rakeb syndrome #606693	*ATP13A2* AR *610513	Mitochondria, lysosome	ATPase that transports inorganic cations and other substrates across cell membranes	Juvenile onset parkinsonism, dystonia, supranuclear gaze palsy	GP, putamen	Cerebral, cerebellar and brainstem atrophy.	Unknown	

Neuroferritinopathy #606159	*FTL* AD *134790	Cytosol	Subunit of the intracellular iron storage protein ferritin	Adult-onset chorea, tremor, dystonia. May have respiratory chain complex assay defects.	GP, putamen, caudate nucleus, thalamus, dentate	BG necrosis, mild cerebral and cerebellar atrophy.	Basal ganglia cavitation and numerous iron-positive inclusions in GP and putamen	

Woodhouse-Sakati syndrome #241080	*DCAF17* AR *612515	Nucleolus	Nucleolar protein with possible substrate receptor for CUL4-DDB1 E3 ubiquitin-protein ligase complex	Dystonia, hypogonadism, diabetes mellitus, alopecia	GP	Prominent white matter T2 hyperintensity, cerebellar atrophy	Unknown	

CoPAN #615643	*COASY* AR *609855	Mitochondria	Enzyme in CoA biosynthesis	Childhood onset motor impairment, dystonia, spasticity	GP, SN	GP calcification	Unknown	


Iron accumulation within the brain is both a recognized part of the normal ageing process and observed in the brains of people with common neurodegenerative diseases, including Alzheimer and Parkinson diseases [12]. NBIA disorders were originally described and grouped based on post-mortem accumulation of brain iron initially identified by gross pallidal discoloration and later by microscopic imaging using Perl’s stain, Turnbull blue, or ferritin immunostaining [13]. The distribution of excess iron accumulation in NBIA disorders echoes the normal regional distribution of iron within the brain with the most iron-rich regions being (in decreasing order) the globus pallidus, red nucleus, putamen, substantia nigra, and the caudate nucleus [14].

Transferrin is the primary iron transport protein in the brain, with brain iron homeostasis mediated by iron uptake across the blood-brain barrier by endothelial cells, subsequent endocytosis of transferrin by neurons, and export via ferroportin [15]. Caeruloplasmin is required to oxidize ferrous iron, and ferritin, comprised of light (encoded by *FTL*) and heavy chains, is the primary intracellular iron storage protein [12].

Drecourt et al. [16] propose that dysfunction in several NBIA genes leads to inappropriately normal transferrin receptor (TfR1) level even in the presence of iron overload. They link this to impaired recycling of TfR1 due to impaired post-transcriptional regulation by TfR1 palmitoylation leading to iron accumulation, potentially as the unifying step linking the mutated genes in the studied fibroblasts (with variants in *PANK2, PLA2G6, FA2H, C19orf12, REPS1*, and *CRAT*) [16]. While this hypothesis may in part explain the iron accumulation seen in these and other disorders, it has not been shown that iron dyshomeostasis directly causes neuronal death nor does it explain why the observed neurodegeneration preferentially affects specific brain regions – this may be related to gene expression patterns, differential iron susceptibility of neuronal subtypes, or reflect the underlying iron-rich brain regions.

Ferroptosis is a recently described mode of regulated cell death, separate to apoptosis or autophagy, that may provide an alternative explanation for the protective role of iron chelation observed in some NBIA models. Ferroptosis is characterized biochemically by accumulation of lipid peroxides and reactive oxygen species derived from iron metabolism with mitochondrial morphological changes and outer membrane rupture [15,17]. Experimentally, it can be prevented by iron chelation, and ferroptosis sensitive cells have altered expression of key iron homeostasis proteins (TfR1 increased, ferritin decreased) implying that cellular iron overload contributes [17]. However, to date ferroptosis has not been directly linked to any NBIA [15].

Irrespective of the mechanisms underpinning iron accumulation, it is not surprising that iron chelating agents have been historically explored as potential treatments for NBIA disorders [18].

## Childhood NBIA Disorders: Genetics, pathophysiology, and clinical features

Despite genetic heterogeneity, the different NBIA disorders share several common features. The four most common disorders presenting in childhood are detailed below and the remaining are summarized in ***Table 2***.

### PKAN

PKAN is caused by disruption to the co-enzyme A (CoA) biosynthesis pathway from vitamin B5 (***Figure 1***) due to biallelic loss-of-function variants in *PANK2* [19,20,21]. While reduced CoA levels are not apparent on testing [22], disruption of CoA biosynthesis is postulated to lead to a reduction in CoA available specifically for phosphopantetheinylation of a number of proteins where this is needed for post-translational activation, and possibly reduction of CoA and acetyl-CoA available as a cofactor or substrate for a large proportion of metabolic reactions [23]. Proteins activated by phosphopantetheinylation include the mitochondrial acyl carrier protein (mtACP) involved in mitochondrial fatty acid synthesis, α-amino adipic semialdehyde synthase (AASS) in lysine metabolism, and 10-formyltetrahydrofolate dehydrogenase (10-FTHFDH) involved in folate metabolism [24]. Observed effects seen in PKAN cellular and animal models include disrupted iron homeostasis, impaired dopamine metabolism, reduced mitochondrial respiration, and impaired mitochondrial fatty acid synthesis [21,23].

PKAN arises only through loss of PANK2 function, despite retaining normal function of isoenzymes PANK1 (alpha and beta isoforms), and PANK3, which appear to be expressed throughout the brain, though all isoforms are expressed at relatively lower levels in the basal ganglia (GTEx) [25]. *PANK2* is the only isoform containing a mitochondrial targeting signal, which localizes it to the mitochondria. It is unclear why the other PANK isoforms are unable to rescue mitochondrial function by translocation of intermediary metabolites of CoA, such as 4’-phosphopantetheine which is membrane permeable, that should be present in other cellular compartments [26,27]. One possible explanation is that activation of mtACP, which consumes CoA, may require a reserve of CoA within the mitochondria that is diminished with loss of PANK2 function [21,23].

PKAN presents often insidiously with clumsiness, gait difficulties or subtle loss of motor skills, with an ensuing movement disorder characterized by severe generalized dystonia and later parkinsonism, with loss of vision due to retinal degeneration. Onset in classic PKAN is in the first decade and progresses to severe movement disorder including recurrent status dystonicus and premature death; the atypical form presents later and progresses more slowly. The archetypal finding of the ‘eye of the tiger’ appearance on MR brain imaging is caused by the combination of tissue edema or gliosis and exquisitely localized iron deposition in the globus pallidus [28]. Typical pathological findings in post-mortem analysis of PKAN show focal globus pallidus neuronal loss, gliosis, iron deposition, and spheroidal structures derived from degenerating neurons [29,30]. The focality of these changes in PKAN is still not adequately explained, given ubiquitous brain *PANK2* expression and the central role of CoA in numerous biochemical reactions.

### PLAN

PLAN is caused by biallelic pathogenic variants in *PLA2G6*, which encodes a phospholipase A2 enzyme that catalyzes the breakdown of phospholipids into fatty acids [31,32]. This has a central role in membrane remodeling, regulation of apoptosis and possible roles in cellular signaling and regulation [32]. PLA2G6 is found throughout the cell including the mitochondria, with abnormal mitochondrial physiology and increased lipid peroxidation reported in PLAN [33].

PLAN encompasses a spectrum of clinical disorders associated with cognitive and motor regression, movement disorders (including spasticity, axial/limb dystonia and ataxia), and bulbar dysfunction. This ranges from the classical and most severe form, infantile neuroaxonal dystrophy (INAD), to atypical neuroaxonal dystrophies (aNAD) which may present from early childhood up to early adulthood and has more gradual progression. A third phenotype is the typically adult-onset *PLA2G6*-related dystonia-parkinsonism [34]. Neuropathology findings demonstrate widespread axonal spheroids, enlarged abnormal mitochondria, iron deposition, and presence of Lewy bodies, neurofibrillary tangles and hyperphosphorylated tau [35,36].

### MPAN

MPAN is caused by biallelic or, more rarely, monoallelic mutations to *C19orf12*, which is a small mitochondrial transmembrane protein [37]. Heterozygous pathogenic variants causing autosomal dominant inheritance appear to arise from a dominant-negative effect where nonsense variants in the last exon are predicted to avoid nonsense-mediated decay [38]. Protein function is not well established but is suspected to have a role in lipid homeostasis as it is co-regulated with proteins of fatty-acid biogenesis and mutations lead to mis-localization and mitochondrial dysfunction [37,39].

MPAN is most commonly characterized by childhood-onset gait disturbance, progressive dystonia, pyramidal signs, neuropsychiatric symptoms and cognitive decline; however an adult-onset form is well documented [30,40,41]. Pathological findings are similar to those seen in PKAN, though with the additional finding of widespread Lewy bodies and neurites, with near complete neuronal loss in the substantia nigra. Iron deposition however appears limited primarily to the GP [30,37].

### BPAN

Beta-propeller protein-associated neurodegeneration (BPAN) is caused by hemizygous or heterozygous variants to *WDR45*, arising in an X-linked dominant fashion due to X chromosome inactivation, usually from *de novo* variants [42]. WDR45 has a role in autophagy with defects shown to impair cellular autophagy with subsequent iron overload, reduced ferritin expression, and mitochondrial dysfunction [43,44]. Recently, a *WDR45* mutation has been shown to reduce degradation of the transferrin receptor by impairing autophagy, providing a further link to iron accumulation and neuronal degradation [45].

In contrast to other recessively inherited NBIAs, BPAN has a strong female preponderance with affected males predicted to have post-zygotic mutations and male germline mutations thought to be non-viable [42]. The normally random X chromosome inactivation is commonly skewed in female BPAN patients, preferentially silencing the dysfunctional allele, which, as with the somatic mosaicism in males, may impact on phenotype severity [46]. It is associated with a distinctly recognizable but complex phenotype with early-onset developmental delay, epilepsy, ataxia, learning difficulties, and stereotypical behavior in childhood followed by secondary neurological deterioration with prominent parkinsonism and dystonia in early-adulthood [47]. Brain MR imaging shows T2-weighted signal hypointensity in the GP and SN due to high iron, a distinctive T1-weighted hyperintense ‘halo’ in the midbrain, and often cerebral atrophy [47]. There are associated pathological findings of gliosis, iron deposition, neuronal loss with axonal spheroids, and tau pathology [48].

### Other

Rarer but established NBIA disorders are included in ***Table 2***: Kufor-Rakeb syndrome (*ATP13A2)* [49], neuroferritinopathy (*FTL*) [50,51], aceruloplasminemia (*CP*) [52], Woodhouse-Sakati syndrome (*DCAF17*) [53], and CoPAN (*COASY*) [54,55]. Isolated clinical cases have been described with clinical and radiological features of NBIA with associated single gene variants, though with limited clinical or molecular genetic information or lack of corroboration at present: *SCP2* [56], *GTPBP2* [57,58], *AP4M1* [59], *CRAT* [16], and *REPS1* [16].

## Therapeutic approaches for NBIA disorders

Therapeutic approaches include generic treatments such as iron chelation, aimed at reducing brain iron accumulation, and deep brain stimulation as palliation for intractable dystonia. Increasingly there is a move towards disease-specific approaches or strategies targeted at correcting the underlying gene or enzyme defect. Currently under evaluation include the use of small molecules for substrate supplementation to either bypass disrupted enzymes or enhance enzyme activity in residual functional proteins or their unaffected isoforms. Other strategies undergoing pre-clinical evaluation involve precision therapies to repair or replace defective enzyme function either through enzyme replacement, or targeted gene therapy.

### Iron chelation therapy

Iron chelation therapy has been investigated as a potential generic approach to treat NBIA, irrespective of the underlying etiology. There is good reason to suspect that iron accumulation is part of the pathological process leading to neurodegeneration in the NBIAs, given the essential role that iron plays as an enzyme cofactor and within respiratory chain metalloproteins, and the noted iron accumulation in multiple neurodegenerative diseases including Parkinson’s disease [60]. It has thus been hypothesized, that although iron metabolism is not the primary pathway disrupted in the majority of NBIA disorders, reducing iron load could potentially decelerate neurodegeneration. As a result, the iron chelator deferiprone has been trialed in PKAN and other NBIAs.

#### Deferiprone

Deferiprone is an oral iron chelator, originally utilized for iron overload secondary to blood transfusions given for hematological disorders such as thalassemia [61]. As it is able to cross the blood-brain barrier and retrieve iron which is subsequently excreted, it was proposed to have clinical potential through repurposing for patients with NBIA [62]. Initial case reports of use in PKAN [63,64] suggested a potential benefit, though two reported uses in BPAN [65,66] were associated with either no change or worsening of parkinsonism; this was followed by three open-label pilot trials in small numbers of patients with PKAN which provided evidence that brain iron deposition was reduced on serial MRI scans [62,67,68,69]. Open-label case-series including a total of 48 patients have been published on iron chelation in aceruloplasminemia though without convincing benefit shown to neurological progression [70], and an open-label pilot clinical trial is currently recruiting studying deferiprone treatment in aceruloplasminemia (NCT04184453).

The first clinical trial from the TIRCON (Treat Iron Related Childhood-Onset Neurodegeneration) collaboration was an 18-month, multi-center, randomized, placebo-controlled, double-blind trial which assessed the safety and efficacy of deferiprone in 88 individuals (41 classic, 45 atypical) with PKAN with an 18-month open-label extension [71]. The primary outcome measures were the Barry-Albright Dystonia (BAD) scale and Patient Global Impression of Improvement (PGI-I) scale.

Deferiprone was well tolerated and safe, with anemia (requiring iron supplementation) being the only treatment-emergent adverse event, occurring in 12/58 of the treatment group. It significantly reduced iron accumulation on quantitative serial MRI assessment in a subset of participants. After the 18-month placebo phase there was a non-significant difference in BAD score between active and control groups (difference between groups of –1.51 points, 95% CI –3.19 to 0.16), but in the 18-month open-label extension there appeared to be a slowing of decline in those that switched from placebo (4.4 points on placebo, 1.4 points on active, p = 0.021). In addition, planned subgroup analysis of atypical PKAN patients showed a smaller worsening of their BAD score compared to placebo (mean difference –2.19, p = 0.019). No difference was detected by patients either between active and control groups, or in those that switched from placebo to active as assessed by the PGI-I scale and there was only weak correlation between the PGI-I and BAD scores [71].

The authors of the study noted the slower than anticipated reduction in BAD scores in the placebo group, coupled with the lack of a good natural history study for PKAN, led to an underpowered study design which may have reduced the strength of associations seen. However, without the realistic prospect of enrolling significantly more patients with such a rare disorder, the alternative may be to extend trial design and focus on enrolling less severely affected individuals earlier in the disease course to avoid a scale attenuation effect in any outcome measures for future studies. This should be coupled with a better understanding of the natural history of these disorders to identify more sensitive markers of disease progression, as should be attained through current observational studies (TIRCON registry and NBIAready natural history study (NCT02587858)) [72].

### Palliative and symptomatic approaches

#### Deep brain stimulation

Surgical treatments have proven to be efficacious for a number of the NBIAs. Although these are by nature palliative, they can bring marked symptomatic improvement and can be targeted and individualized. Deep brain stimulation (DBS) uses electrodes placed deep into the grey matter structures of the brain with stimulation controlled by a subcutaneous pacemaker-like device. Stimulation can be targeted to various structures and modulated according to response.

There are a number of case series reporting outcomes after DBS in NBIA [73,74], typically using the Burke–Fahn–Marsden Dystonia Rating Scale Motor Score (BFMMS) as primary outcome, with the largest retrospective data set coming from Timmermann at al. in 2010 [75]. They reported 23 NBIA patients aged 6-36 at the time of surgery, though not all were genetically confirmed to have PKAN, with an overall good response to bilateral globus pallidus internus (GPi) DBS insertion, as measured by dystonia scales and quality of life reporting [75]. In another cohort containing 13 NBIA patients, Lumsden et al. report significant improvement in dystonia rating scale scores at 6 months post GPi DBS, although the statistical significance did not persist at 12 month review [76]. This early response may be typical for children with classical PKAN, whereas adolescents or young adults with atypical PKAN seem to have longer-lasting benefits. A recent meta-analysis of 26 reported PKAN patients confirmed this short term improvement in movements (BFMMS 27%, –21.4 improvement), and although this did not distinguish between classic or atypical disease, the mean age at dystonia onset (12.6 years) suggests most would be classed as atypical PKAN [77].

Most of these reports refer to PKAN, though there are case rare reports of DBS use in others such as PLAN [78] where it has been used as emergent treatment of dystonic crisis to good effect and *DCAF-17* related dystonia with improvement on rating scales at 12 and 18 months post-surgery [77].

#### Thalamotomy and pallidotomy

For intractable severe dystonia, six PKAN cases were reported to have had either stereotactic surgical pallidotomy [79,80], thalamotomy [81,82], or combined pallidothalamotomy [83] with improvement in limb function and pain. Pallidotomy is reported to be similarly efficacious to DBS in management of intractable dystonia in larger case series of diverse etiologies, without the risk of complications related to hardware failure [80,84]. However, the suitability of these irreversible procedures and risks of bilateral ablation when neuromodulatory approaches are available (and not contraindicated) is not clear.

#### Intrathecal or intraventricular baclofen

Intrathecal Baclofen (ITB) administration from an abdominally placed continuous pump is an established palliative therapy for treatment-resistant spasticity and dystonia and is often recommended for NBIAs [85]. However, reports of outcomes in NBIAs from ITB is limited to seven cases of its use in PKAN patients with carer-reported improvement in dystonia and ease of care but without evidence from validated scales [86,87,88,89]. Intraventricular baclofen administration is also reported in a single child with PKAN which significantly improved his dystonia, but the device had to be removed after 5-weeks due to pump infection [90].

#### Botulinum toxin

Intramuscular injection of botulinum toxin has been used as a palliative approach to manage severe dystonia more generally, though there are limited reports of use and efficacy in NBIAs [91,92,93]. A retrospective description of palliative care in eight patients with PKAN and one with MPAN reported that botulinum toxin was effective at reducing dystonia, spasticity, salivation, and pain while aiding caregiving [94].

#### Other

A case report of the use of continuous intrathecal infusion of morphine for intractable dystonia in a 14-year old patient with PKAN showed improvement in motor function and reduction in dystonia [95].

### Small molecule treatments

Targeted small molecule approaches to NBIA treatment are by their nature disease-specific. For these primarily recessive monogenic disorders, where the protein function is known, treatment aims to bypass blocks in the pathway or reverse the downstream effects of protein dysfunction. This is clearest in the NBIAs that disrupt CoA synthesis such as PKAN, where treatment approaches to date have attempted to bypass the lack of PANK2 enzyme activity by providing a downstream metabolite, or pro-metabolite further along the pathway (***Figure 1***). The only randomized controlled trial reported to date for this approach assessed safety, tolerability and short-term efficacy of fosmetpantotenate, a synthetic precursor for the endogenous intermediate 4’-phosphopantothenate. Trials are currently underway to assess oral dosing of 4’-phosphopantetheine (4’-PPT), an endogenous intermediate metabolite further downstream in the CoA synthetic pathway. An alternative approach in pre-clinical development is the use of small molecules to augment the activity of alternative isoforms of the PANK enzymes, PANK1 and PANK3, using a class of drugs referred to as pantazines [96].

The recent finding of reduced TfR1 palmitoylation in several cellular NBIA models suggests inducers of palmitoylation as a potential treatment for several NBIAs. Indeed Drecourt et al. report that artesunate, an antimalarial drug in current use, is able to rescue the abnormal palmitoylation in fibroblasts [16].

### PKAN

#### Pantothenate

Pantothenate, commonly known as vitamin B5, was the first small molecule therapy proposed for PKAN on the basis that either residual PANK2 function could be increased by increasing substrate concentration, or that this might increase availability of downstream metabolites from other PANK isoforms [19]. This has never been assessed in a clinical trial, though there are patient-reported benefits and consensus guidelines continue to recommend a trial of pantothenate particularly for atypical PKAN in which there is hypothesized to be higher residual enzyme function [85].

#### Fosmetpantotenate

Fosmetpantotenate is a 4’-phosphopantothenate precursor designed to be membrane-permeable, allowing it to be dosed enterally and cross the blood-brain barrier, where once taken up into cells, it would be metabolized to 4’-phosphopantothenate and serve as a substrate for CoA generation (***Figure 1***) [97].

After an initial case report of open-label use in a single young adult [98], fosmetpantotenate was assessed in the recently reported FORT trial, a multinational, randomized, placebo-controlled, double-blind study with an open-label extension [99]. The primary outcome of the FORT trial was change in patient-reported PKAN Activities of Daily Living (PKAN-ADL) scale, with secondary endpoints of the BAD scale and a number of others. 84 patients were enrolled and 78 completed the trial with reasonable compliance. Unfortunately there was no indication of efficacy in the pre-specified outcome measures over the 24 week placebo-controlled phase, and further development of the compound was halted [99].

This negative result may be due to the outcome measures being insensitive to change over the 6-month period studied – the PKAN-ADL score improved by 1.4 points (6.6% of baseline) in the placebo group, perhaps reflecting fluctuating disease or placebo effect. Alternatively, there may have been lack of target delivery of the drug – pre-clinical experiments suggested that fosmetpantotenate crossed the blood-brain barrier when orally dosed in monkeys though not mice, but this has not been proven in humans. Finally, given the mitochondrial localization of PANK2 it is likely the drug would need to reach the mitochondria or supplement cytosolic phosphopantothenate sufficiently to induce translocation, which is not proven in the published pre-clinical data [97].

#### Pantethine

Pantethine is a licensed drug for the treatment of hyperlipidemia and was repurposed as a potential PKAN treatment, providing an alternative substrate to pantetheine to form 4’-phosphopantetheine [100]. Pre-clinical assessment of pantethine in a fly model suggested that it rescued CoA levels and mitochondrial dysfunction with improved locomotor phenotype when added to food [100]. Similarly, in a zebrafish model addition of pantethine to the water rescued the observed phenotype [101]. In a mouse model that combined *Pank2* knock-out with the ketogenic diet to induce a motor phenotype, pantethine added to the diet was able to prevent onset of the motor phenotype and weight loss, mitochondrial changes, and neuropathological changes showing neurodegeneration [102]. There is no pre-clinical data beyond this mouse model to suggest that this molecule will reach the target tissue in humans, and a critical limitation of this study was the alternate possibility that metabolism of pantethine to pantothenate would also have protected animals. Given the presence of pantetheinases in mammalian circulation, the likelihood that the pantethine molecule remains intact *in vivo* is very low. However, there are long term safety data as it has been in use since the 1980s for hyperlipidemia.

A recently reported pilot trial tested daily oral pantethine in a single-arm, open-label trial in 15 children (aged 4–13) with PKAN in China (ChiCTR1900021076) [103]. Primary outcome measures were the change in UPDRS and Burke-Fahn-Marsden scores after 24 weeks of treatment, with a secondary endpoint comparing the rate of change to the previous 24 weeks. They found no change in either score over the trial period, though they reported a slowing in score progression when compared to the pre-trial period. Safety analysis, limited to two timepoints over the trial, did not report significant side effects. Serum CoA levels were assessed and were unchanged, as would be expected as this biomarker has not been shown to vary with disease state in PKAN.

#### 4’-phosphopantetheine

4’-phosphopantetheine (4’-PPT) is an endogenous intermediate metabolite in the vitamin B5-CoA pathway (***Figure 1***) currently being evaluated as a treatment for PKAN by bypassing the dysfunctional PANK2 enzyme. Srinivasan et al. [104] identified 4’-PPT as an extracellular source for CoA synthesis in 2015, contrary to the previous understanding that vitamin B5 was the essential precursor to the pathway. Furthermore 4’-PPT was observed in cells and organisms to be stable and membrane-permeable, allowing passive diffusion [104]. 4’-PPT enteral dosing in a mouse model of PKAN (*Pank2* knock-out) corrected abnormalities in iron homeostasis, mitochondrial function and dopamine metabolism in the globus pallidus [21]. A phase 2 randomized, double-blind, placebo-controlled clinical study of 4’-PPT in PKAN is underway in North America (NCT04182763) and in development in the UK and the Netherlands.

#### Pantazines

Pantazines are a class of molecules that have been developed as generic PANK activators (***Figure 1***). Based on the observation that in PKAN, only PANK2 is affected, it is hypothesized that augmentation of isoforms PANK1 or PANK3 might be able to compensate for dysfunctional PANK2 and treat PKAN. PZ-2891 has been identified through high-throughput screening and subsequent validation as a selective activator of PANK3, with pre-clinical studies suggesting it will cross the blood-brain barrier and can increase the CoA content of a cellular and a mouse model with impaired CoA synthesis which shares some features with PKAN [96]. Current work on this and other pantazines is focusing on optimizing the pharmaceutical properties, aiming to bring this to clinical trial in the future.

#### Other

Other approaches in various stages of pre-clinical development for the treatment of PKAN include various pro-drug formulations of metabolic intermediaries, for example, cyclic phosphopantothenic acid which is reported to be stable and membrane permeable in cells and mice [105], and acetyl-4’-phosphopantetheine which is stable in serum and prevents cellular phenotypes [106]. Treatment with coenzyme A would seem an obvious approach; *in vitro* administration of CoA to neurons corrects cellular pathology and addition to the water of a zebrafish model rescues the phenotype, but this has not progressed to treatment as it is likely to be catabolized *in vivo* and may not be membrane permeable [101,107].

### PLAN

#### Deuterated polyunsaturated fatty acids – RT001

Work on a *Drosophila* model of *PLA2G6*-related disease and human PLAN fibroblast lines has shown significantly elevated lipid peroxidation and mitochondrial dysfunction [33]. Deuterated polyunsaturated fatty acids (D-PUFA) that inhibit lipid peroxidation were able to partially rescue the fly model locomotor abilities and mitochondrial membrane potential in patient-derived fibroblasts [33]. As a result, RT001 – a deuterated homologue of linoleic acid which aims to make polyunsaturated fatty acids resistant to lipid peroxidation – has been proposed as a treatment [33]. An anecdotal report of use in two children has been published with some suggestion of efficacy [108], and a single-arm open-label clinical trial in 19 patients is due to have completed but no data have yet been reported (NCT03570931).

#### Desipramine

The *pla2g6*-knockdown fly model has also shown elevation of ceramide levels that accumulate in lysosomes, with subsequent cell membrane and endocytosis defects [109]. Sphingomyelinase converts ceramide phosphoethanolamines and sphingomyelin to ceramide, a process that is inhibited by desipramine, a tricyclic antidepressant used for the treatment of depression. Ceramide reduction in a *Drosophila* null model improved the retinal and startle phenotype [109]. As a result, there was an off-label repurposing of despiramine in an open-label clinical trial of 4 INAD patients (NCT03726996).

### Other NBIAs

Currently there are very few precision therapy options for other monogenic NBIAs. For BPAN mTOR signaling inhibition with rapamycin in a cell model rescued reduced autophagy and reduced apoptosis [110]. Ceruloplasmin replacement therapy by intraperitoneal injection has been demonstrated to be effective in a knock-out mouse model of aceruloplasminemia [111]. A single case is reported where a patient with acerulopasminemia reported benefit with oral zinc sulphate, considered due to reported efficacy in Wilson disease, though it is unclear what impact zinc has on iron metabolism [112].

### Gene therapy

It may be argued that true precision therapy for these monogenic disorders must ultimately focus on correcting the underlying genetic abnormality in target cells within a therapeutic window that precedes significant irreversible neurodegeneration. Advances in genetic therapies are now rapidly translating into clinical practice for children with neurological and neuromuscular disorders. Emerging technologies with potential for translation into clinical practice include antisense oligonucleotide (ASO) therapy, adeno-associated virus (AAV) vector gene delivery, and genome editing using the bacterial CRISPR-Cas9 system.

#### Approaches

ASOs are short single-stranded DNA molecules designed to be complimentary to target mRNAs which can be used to modulate protein expression or induce changes in splicing (exon inclusion or skipping). Nusinersen, an ASO that causes integration of exon 7 into *SMN2* mRNA enhancing full-length SMN protein expression in patients with spinal muscular atrophy (SMA), is now licensed for clinical use in a number of countries worldwide. Target delivery of ASOs is challenging in neurological disease, though following advances in ASO technology, similar exon-skipping for SMA patients is hoped for from the orally deliverable risdiplam [9]. Delivery of ASOs also needs to be repeated — for example, through regular lumbar puncture for intrathecal delivery – as they do not genomically integrate into patient DNA. Furthermore, for many diseases, one specific ASO cannot be used to treat the entire patient population because they need to be tailored to the specific genetic variant, as with the multiple different exon-skipping ASOs for Duchenne muscular dystrophy or the recent report of a splice-modulating ASO developed for a single child with neuronal ceroid lipofuscinosis 7 [113].

AAV vector gene delivery was first utilized in the 1980s, though only more recently has it come to the bedside. This exploits the virus’ ability to penetrate human cells and release the contained DNA strands to deliver therapeutic genes to the nucleus for expression. AAV vectors integrate only at low levels into the target cell genome, and largely exist as extra-circularized DNA that can persist for years [114]. AAV vector-delivered gene therapy is now an established and licensed treatment for SMA with the recent approval of onasemnogene abeparvovec, which is an intravenous treatment that delivers a working copy of human *SMN1* to motor neurons using AAV9 [115]. Challenges to this approach for neurological disorders again include achieving target cell integration and correct brain biodistribution, factors that potentially require a targeted neurosurgical approach. Such neurosurgical targeting has proven effective in aromatic L-amino acid decarboxylase (AADC) deficiency, where a recombinant AAV cassette containing a working copy of defective enzyme L-dopa decarboxylase is stereotactically injected into bilateral putamina or substantia nigra pars compacta [10,116].

Genome editing is now an established laboratory tool using the CRISPR-Cas9 system which uses guide RNA to locate DNA sequences, with subsequent advances allowing single-base editing or precise integration of desired sequences. This most recent advance, called prime editing, holds promise for neurological disorders such as NBIAs, as it has increased editing efficiency and can be used on post-mitotic, non-dividing cells such as neurons [117]. However, these approaches are not yet mature for clinical translation.

#### Application of novel genetic therapies to NBIA disorders

The monogenic NBIA disorders should be amenable to a number of available gene therapy approaches.

While ASO therapies are less suitable to autosomal recessive loss-of-function disorders, in autosomal dominant MPAN patients, ASOs could potentially knock down the mutant allele, which is hypothesized to multimerize with the wild-type allele and cause its failure and restore protein function. For PKAN and other recessive NBIA disorders in which variants are predicted to cause improper splicing, ASOs may be able to restore function.

AAV vector-mediated delivery of functional genes is potentially an attractive option for a number of NBIA disorders in that this approach can restore gene expression in loss-of-function recessive disorders, and targeted putaminal and midbrain AAV9 delivery is already established [11,10]. To date, gene replacement therapy using an AAV vector has been administered in *plag2g6-inad* mice in the neonatal period prior to onset of symptoms, and was successful in rescuing motor symptoms and neuropathological features [118]. Following this proof-of-concept study, further preclinical toxicology and biodistribution studies will drive future clinical translation to PLAN patients. Genome editing is the ultimate panacea of targeted precision medicine and once established should be eminently suitable for these disorders.

#### Challenges

Despite recent advances, the NBIAs pose significant challenges to pre-clinical gene therapy development. While there are some NBIA animal models (typically *Drosophila*, zebrafish, or mouse) that show motor deficits reminiscent of the human phenotype, others such as the PKAN mouse model [119] do not perfectly recapitulate the motor or cognitive features of human disease, thereby limiting their utility in assessing the true efficacy of gene therapy in the murine model. This may be somewhat circumvented by monitoring target cell biomarkers that recapitulate key features of the human disease, such as those used by Jeong et al. to demonstrate efficacy of 4’-PPT in the same PKAN model [21]. Furthermore, there is a paucity of suitable large animal and primate models for pre-clinical biodistribution and efficacy studies before human trials. Serendipitous identification of large animal models such as sheep with PLAN [120] may help short-cut this progress, and genetic screening of animals in primate research facilities to identify rare heterozygous variants may help further.

By definition, NBIA disorders affect the CNS and require treatments that bypass the blood-brain barrier either intrinsically or by intrathecal, intraventricular, or targeted intraparenchymal delivery. These approaches can be technically challenging but may also reduce off-target effects. While some NBIAs, like PKAN, seem to be exquisitely focal in their anatomical localization and cellular dysfunction suggesting targeted GP delivery may be the best approach, for most the dysfunction is likely to be widely distributed though the CNS. For these, various delivery routes of various AAV vectors are under investigation with intrathalamic, intracerebroventricular or cisterna magna delivery showing promise in animal models [121,122].

## Conclusion

Although targeted therapies in NBIAs remain very limited, with no proven disease-modifying treatments, there is a growing paradigm shift towards precision medicine for these disorders. A number of clinical obstacles still need to be overcome to facilitate treatment development in the NBIAs. All are ultra-rare disorders (prevalence for each is estimated to be under 1 per million population) in which delay in clinical recognition will often lead to significant disease progression prior to diagnosis [123]. Neuronal loss is thought to be largely irreversible, so early intervention is likely needed for the best clinical outcomes within a ‘therapeutic window’. Furthermore, the lack of readily available robust biomarkers limits the objective measure of therapeutic efficacy.

Despite these challenges, there continues to be progress towards targeted therapies. Improving access to rapid whole exome or whole genome sequencing should reduce the time to diagnosis, and there are now several international registries collecting data necessary to develop relevant outcome measures for future clinical trials. The completed clinical trials in PKAN are prime examples of international collaboration bringing together patient cohorts of sufficient size for randomized, controlled clinical trials; however, power to detect improvement is limited by the available clinical outcome measures and lack of relevant and useful disease-specific biomarkers. Advances in quantitative MRI and translational omics should facilitate future development of NBIA biomarkers for diagnosis, disease monitoring and assessment of drug efficacy.

Improved understanding of disease mechanisms continues to reveal novel treatment targets, and high-throughput drug screening using established models is showing promise, for example with the identification of the pantazines. Recent advances in other neurological disorders, some similarly rare such as AADC deficiency, gives hope that gene therapy is both scientifically and financially feasible for these conditions.

It is hoped that by addressing these challenges, the development of novel NBIA precision therapies will be accelerated in the foreseeable future, to better treat these medically resistant neurodegenerative disorders, with the ultimate goal of reducing mortality risk and increasing quality of life for patients and their carers.

## Additional File

The additional file for this article can be found as follows:

10.5334/tohm.661.s1 Supplementary Table 1. Literature research results, PubMed (*https://pubmed.ncbi.nlm.nih.gov/*), April 2021.
