# Supplementary material for: Towards Precision Therapies for Inherited Disorders of Neurodegeneration with Brain Iron Accumulation
Source: Tremor Other Hyperkinet Mov (N Y). 2021 Nov 24;11:51. doi: 10.5334/tohm.661 (PMC8641530; doi:10.5334/tohm.661)
Supplement: Supplementary Table 1. — Literature research results, PubMed (https://pubmed.ncbi.nlm.nih.gov/), April 2021. [file tohm-11-1-661-s1.pdf]

Towards precision therapies for inherited disorders of neurodegeneration with brain iron accumulation

Supplementary information

Search term	Results	Dupli-cates	Non-English	Unique
“Neurodegeneration with Brain Iron Accumulation” AND (“treatment” OR “therapy”)	129	-	3	126
“Pantothenate Kinase-Associated Neurodegeneration” AND (“treatment” OR “therapy”)	188	44	19	125
“PLA2G6-Associated Neurodegeneration” OR “PhosphoLipase A_2-Associated Neurodegeneration” AND (“treatment” OR “therapy”)	12	7	0	5
“Mitochondrial membrane Protein-Associated Neurodegeneration” AND (“treatment” OR “therapy”)	6	6	0	0
“Beta-propeller Protein-Associated Neurodegeneration” AND (“treatment” OR “therapy”)	16	9	1	6
“Fatty Acid Hydroxylase-associated Neurodegeneration” AND (“treatment” OR “therapy”)	1	1	0	0
Aceruloplasminemia AND (“treatment” OR “therapy”)	55	11	3	41
Neuroferritinopathy AND (“treatment” OR “therapy”)	20	11	4	5
“Kufor Rakeb” AND (“treatment” OR “therapy”)	18	2	1	15
“Woodhouse Sakati” AND (“treatment” OR “therapy”)	3	1	0	3
**Totals**	**448**			**326**

Supplementary Table 1 – **Literature research results**, PubMed (https://pubmed.ncbi.nlm.nih.gov/), April 2021.
